# Dataset of the density, water absorption and compressive strength of lateritic earth moist concrete

**DOI:** 10.1016/j.dib.2018.07.032

**Published:** 2018-07-19

**Authors:** Olumoyewa Dotun Atoyebi, Abayomi Emmanuel Modupe, Oluwasegun J. Aladegboye, Sheyanu Valentine Odeyemi

**Affiliations:** Department of Civil Engineering, Landmark University, Omuaran, Kwara State, Nigeria

## Abstract

In this data article, the experimental data of the density, water absorption and compressive strength of lateritic earth moist concrete are presented. 10% and 20% of fine aggregate were replaced with laterite. Potable water at 0.3 water/cement ratio was used and ordinary Portland cement used as the binder. Concrete cubes (150mm×150mm×150mm) were cured and tested at 7 days, 14 days, 21 days, 28 days and 56 days. The reported original data is made publicly available for ensuring critical or extended analyses.

**Specifications Table**

**Table:** 

Subject area	*Civil Engineering*
More specific subject area	*Construction Materials, Concrete Technology*
Type of data	*Table, figure*
How data was acquired	*Casting concrete samples in the laboratory and applying compressive load.*
Data format	*Raw*
Experimental factors	*Earth moist concrete*[1]*and replacement for fine aggregate*[2]*was studied in the literature and some grade 20 concrete cubes were casted at 0.3 water/cement ratio.*
Experimental features	*Fine aggregate replaced with laterite to cast concrete cubes and subjected to compressive load.*
Data source location	*Landmark University Concrete Laboratory, Omuaran, Kwara State. Nigeria.*
Data accessibility	*Data are as presented in this article*

**Value of the data**

• The influence of laterite in the density, water absorption and compressive strength of earth moist concrete is presented here.
• The data gives a basis on ways to utilize earth moist concrete in construction.
• The research data could be helpful in further researches on earth moist concrete.

## 1 Data

**Fig. 1:**
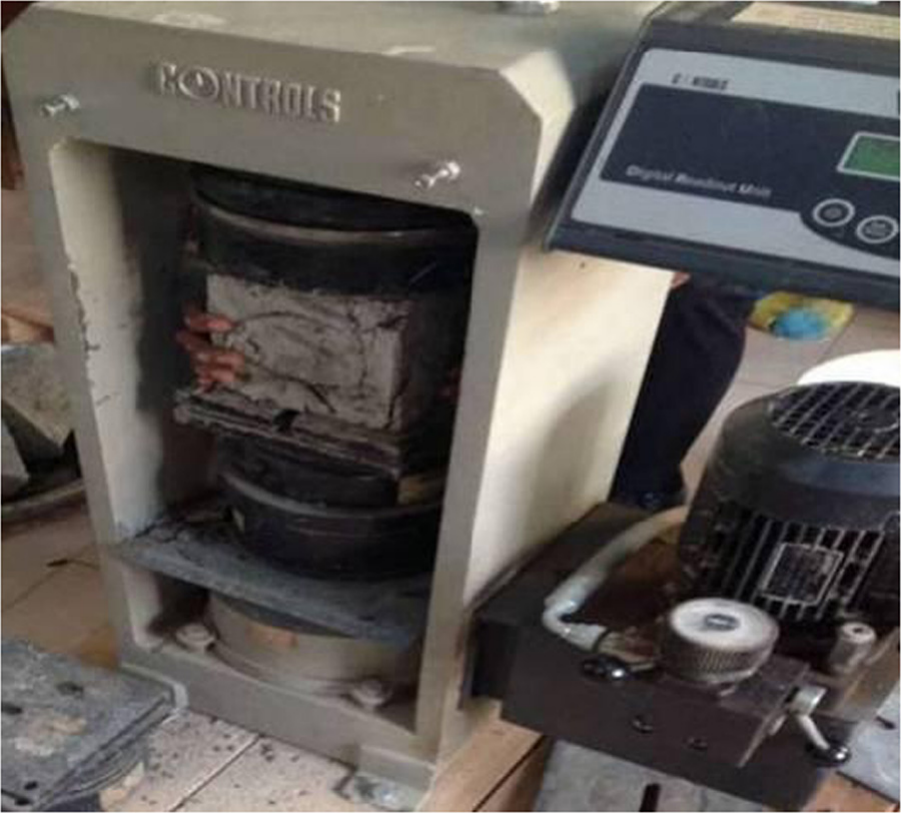
Compressive strength test set up.

**Fig. 2:**
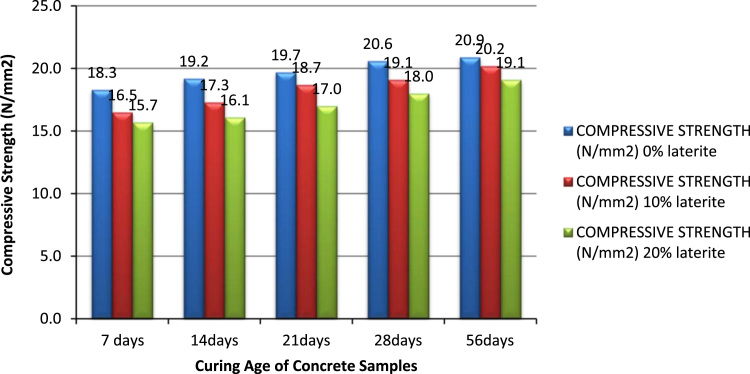
Comparison of compressive strength result of the different replacement samples.

The dataset presented in this article are experimental results of the water absorption and compressive strengths of grade 20 earth moist concrete cubes, fine aggregate was partially replaced with laterite. The tests were carried out at Landmark University Concrete laboratory and the test set up for the compressive strength was shown in Fig. 1. The mean density of the lateritic earth moist concrete cubes were presented in Table 1, Table 2 shows the water absorption value for each concrete cube at different curing age. The compressive strength for the control specimen i.e. earth moist concrete with 0% replacement with laterite was presented in Table 3, Table 4 contains the compressive strength values for 10% replacement at 7 days, 14 days, 21 days, 28 days and 56 days. The results for the 20% replacement are as presented in Table 5. The combined compressive strength result is as shown in Fig. 2.

**Table 1:** Mean density values for all cube specimens at different curing age.

Curing age of specimen	Mean density (kg/m^3^)		
	0% Lateritic content	10% Lateritic content	20% Lateritic content
7 Days	2333.00	2179.00	2145.33
14 Days	2406.15	2206.30	2216.84
21 Days	2287.33	2127.41	2277.28
28 Days	2368.07	2232.37	2239.75
56 Days	2368.64	2148.44	2395.24

**Table 2:** Water absorption values for all cube specimens at different curing age.

Curing age of specimen	Water absorption (%)		
	0% Lateritic content	10% Lateritic content	20% Lateritic content
7 Days	2.40	3.10	3.60
14 Days	2.70	3.28	3.77
21 Days	3.30	3.65	4.17
28 Days	3.40	3.77	4.40
56 Days	3.80	4.30	4.80

**Table 3:** Compressive strength values of Earth moist concrete (0% lateritic replacement) at different curing age.

Curing age of specimen	Mean crushing value (kN)	Compressive Strength (N/mm^2^)
7 Days	412.4	18.3
14 Days	431.7	19.2
21 Days	442.2	19.7
28 Days	463.6	20.6
56 Days	470.3	20.9

**Table 4:** Compressive strength values of Earth moist concrete (10% lateritic replacement) at different curing age.

Curing age of specimen	Mean crushing value (kN)	Compressive strength (N/mm^2^)
7 Days	372	16.5
14 Days	388.2	17.3
21 Days	408.6	18.7
28 Days	429.6	19.1
56 Days	456.1	20.2

**Table 5:** Compressive strength values of Earth moist concrete (20% lateritic replacement) at different curing age.

Curing age of specimen	Mean crushing value (kN)	Compressive strength (N/mm^2^)
7 Days	354.1	15.7
14 Days	362.9	16.1
21 Days	382.9	17
28 Days	404.9	18
56 Days	428.3	19.1

## 2 Experimental design, materials and methods

The concrete aggregate materials were all sourced for in Omuaran, Kwara state, Nigeria. Earth moist concrete with 0.3 water/cement ratio was mixed at 0%, 10% and 20% laterite replacement for the fine aggregate. Concrete cubes (150mm×150mm×150mm) were produced, vibrated, cured and tested at 7 days, 14 days, 21 days, 28 days and 56 days. The cubes were subjected to density test, water absorption test and crushed using the compressive strength testing machine and the values are as presented.
