# Supplementary material for: Dataset of the density, water absorption and compressive strength of lateritic earth moist concrete
Source: Data Brief. 2018 Jul 19;19:2340–3. doi: 10.1016/j.dib.2018.07.032 (PMC6141486; doi:10.1016/j.dib.2018.07.032)
Supplement: Supplementary file 1 — Supplementary material [file mmc1.docx]

Declaration of Interest

This research work is part of a BSc Thesis of Odeyemi Sheyanu Vincent and there is no specific grant from funding agencies in the public, commercial, or non profiting sectors.
